# Massive Pulmonary Embolism Related to a Patient With Heart Failure Secondary to Stress Cardiomyopathy: A Case Report

**DOI:** 10.7759/cureus.52985

**Published:** 2024-01-26

**Authors:** Juan Andres Pimentel-Esparza, Mariana Rios-Gomez, Jorge Antonio Cervantes-Nieto, Juan Alan Fuentes Mendoza

**Affiliations:** 1 Department of Internal Medicine, Petróleos Mexicanos (PEMEX) Regional Hospital Salamanca, Salamanca, MEX; 2 Department of Cardiology, Policlinica Integral del Bajio, Irapuato, MEX

**Keywords:** pulmonary and cardiac pathophysiology, venous thromboembolism (vte), rare case report, takotsubo cardiomyopathy (ttc), pulmonary embolism (pe)

## Abstract

Venous thromboembolism (VTE) is a common disease, which includes deep venous thrombosis (DVT) and pulmonary embolism (PE). It is the third most common cardiovascular disorder, affecting predominantly the male elderly population. Stress cardiomyopathy (SC) is a transitorily cardiovascular condition produced after an emotional or physical trigger, and it features signs and symptoms of acute coronary syndrome. Its pathophysiological mechanisms remain unclear, and SC has also been related to critical complications such as heart failure, arrhythmias, left ventricular outflow obstruction, and thromboembolic events. This case report highlights the association of PE and SC that might play a pathophysiological role.

## Introduction

Venous thromboembolism (VTE) is the third most common cardiovascular disorder. It includes deep venous thrombosis (DVT) and pulmonary embolism (PE) [1]. VTE is triggered by three main factors, as described by Virchow in 1856: vascular damage, venous stasis, and hypercoagulability [2]. Stress cardiomyopathy (SC) is a transitorily cardiovascular condition that presents regional wall motion abnormalities at the left ventricular systolic dysfunction. SC has also been related to some critical complications such as thromboembolic events, within their incidence, and its clinical significance has not yet been established [3].

We present a relatively rare case of a patient who presented with SC, which later evolved into a recurrent episode of PE. This case report highlights the association of PE and SC that might play a pathophysiological role. Currently, there is no strong association to suggest that SC is related to PE.

## Case presentation

A 65-year-old female denied significant chronic degenerative diseases, protruding solely with a body mass index (BMI) of 32.1 and a familiar history of thromboembolism events (PE and DVT).

**Figure 1:**
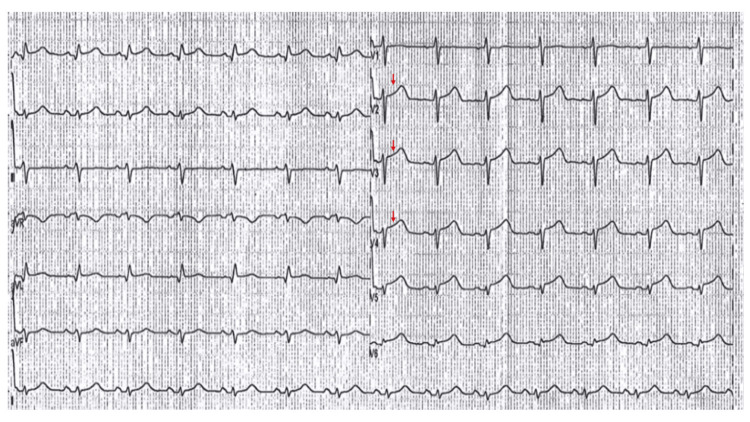
ECG evidence showed ST-elevation segment in the anterior leads.

**Figure 2:**
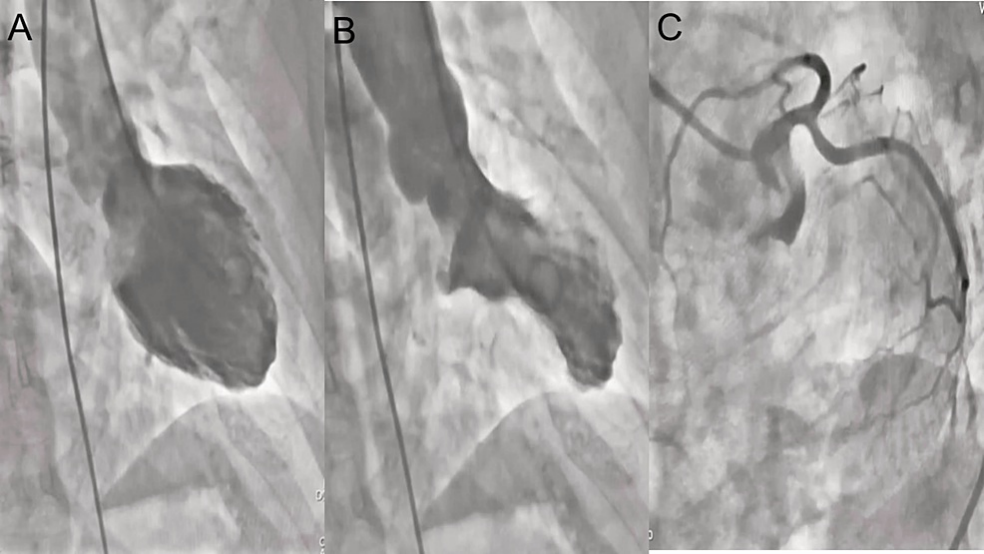
Coronary angiography (CAG) and left ventriculography (LVG). Diastolic phase (A) and systolic phase (B) showed apical dyskinesia. CAG (C) showed normal coronary arteries.

Her first episode began in August 2021. After an emotional event (laboral discussion), she developed an acute onset of chest pain with irradiation to the left arm. She went to the Emergency Department (ED), where the ECG showed ST-segment elevation in anterior leads and augmented vector left (aVL) (Figure 1) with cardiac biomarker elevation (troponin I 24 ng/ml). Fibrinolysis was performed with tenecteplase, without reperfusion evidence. Transthoracic echocardiography (TTE) was performed with evidence of apical, anteroseptal, and anterolateral akinesia with a left ventricular ejection fraction (LVEF) of 67%. Percutaneous transluminal coronary angioplasty (PTCA) demonstrated angiographically normal coronary arteries, and left ventriculography (LVG) showed apical dyskinesia with the octopus pot (Figure 2) and elevation of the end-diastolic pressure of the left ventricle. The patient was discharged after one week with treatment-based beta-blockers (bisoprolol 1.25 mg QD).

In November 2021, acute onset of dyspnea New York Heart Association (NYHA) functional class IV, tachycardia, tachypnea, hypotension, and syncope were presented, causing another arrival to the ED with elevated biomarkers (dimer D 1598 ng/ml, troponin I 7.8 ng/ml, and BNP 281 pg/ml), due to deterioration of clinical conditions. Supplemental oxygen requirement and double inotropic medication (dobutamine and norepinephrine) were initiated, and a computed tomography pulmonary angiography (CTPA) was taken with evidence of PE at the level of the bifurcation of the pulmonary artery. Fibrinolysis was performed with alteplase, and she was admitted to the Intensive Care Unit, where she was treated to recovery. The patient was discharged with symptomatic improvement after one week of admission, with an NYHA class II, and outpatient follow-up posthospitalization was scheduled with a direct oral anticoagulant (DOAC) as rivaroxaban 20 mg QD.

In the follow-up, diagnostic laboratories were requested for a diagnostic approach of hematological and rheumatological etiology, which were found to be within normal parameters (Table 1). So, after one year, due to their adequate evolution, and once these pathologies were ruled out, it was decided that anticoagulant management would be withdrawn.

**Table 1:** Diagnostic laboratories

	Results	Reference Value
Prothrombin time	21.6	12.4–14.9 seg
Partial thromboplastin time	40.2	30.7–37.5 seg
International normalized ratio (INR)	1.64	
Fibrinogen	335	200–400 mg/dl
Antithrombin III	105	80–120%
Protein S	68	60–140%
Protein C	102	60–140%
Factor VIII	104	50–150%
Complement C4	22.90	16–38 mg/dl
Complement C3	97.70	79–152 mg/dl
Rheumatoid factor	<20	< 20 U/ml
Anti-neutrophil cytoplasmic antibodies (c-ANCA PR3)	6.91	Negative ≤ 15 U/ml
Anti-neutrophil cytoplasmic antibodies (p-ANCA MPO)	2.64	Negative ≤ 15 U/ml
Antinuclear antibodies (ANAs)	Speckled 1:80; cytoplasmic 1:40	Negative
Anti-double stranded DNA (anti-dsDNA)	56.22	Negative 0-200 U/ml
Anti-Smith antibodies (anti-Sm)	3.51	Negative < 20 U/ml
Anti-topoisomerase I (anti-Scl-70)	5.93	Negative < 20 U/ml
Anti-nuclear ribonucleoprotein antibodies (anti-nRNP)	2.84	Negative < 20 U/ml
Anti-Jo-1	4.07	Negative < 20 U/ml
Anti–Sjögren's-syndrome-related antigen A antibodies (anti-Ro/SSA)	3.23	Negative < 20 U/ml
Anti-Sjögren's syndrome type B antibodies (anti-La/SSB)	2.57	Negative < 20 U/ml
Anti–cyclic citrullinated peptide (anti-CCP)	0.5	Negative < 5.0 U/ml
IgM anticardiolipin	18	Indeterminate < 20 m MPL/ml
IgG anticardiolipin	3.84	Negative < 15 GPL/ml
IgA anticardiolipin	7.38	Negative < 12 APL/ml

**Figure 3:**
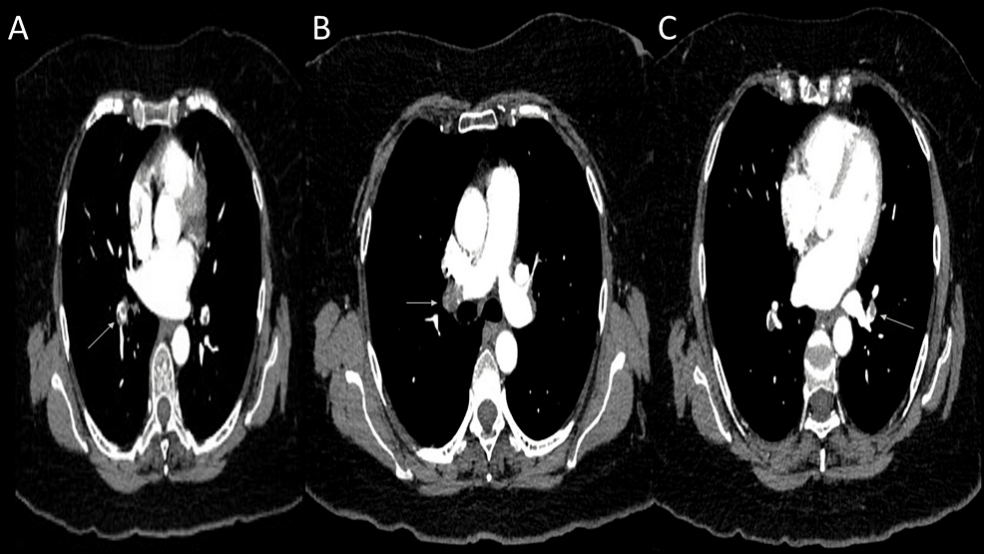
Eccentric central partial repletion at the posterior-basal segment of the right pulmonary artery (A, B) and anterobasal and latero-basal segments of the left pulmonary artery (C).

After one month of suspension of medications, in January 2023, the patient presented with a new episode of acute onset of dyspnea NYHA class III and tachypnea, arriving to the ED with an elevated D-dimer of 9480 ng/ml and troponin I of <0.05 ng/ml. TTE was performed within normal parameters. CTPA evidence showed PE at the posterior-basal segment (Figure 3A) of the right pulmonary artery (Figure 3B) and anterobasal and latero-basal segments of the left pulmonary artery (Figure 3C). Anticoagulation with enoxaparin was reinitiated. The patient was discharged with symptomatic improvement after five days of admission, with an NYHA class II, restarting anticoagulation with DOACs (rivaroxaban 20 mg QD) and supplemental oxygen requirement to maintain oxygen saturation greater than 92%. An outpatient follow-up posthospitalization was scheduled.

## Discussion

VTE is a common disease, which includes DVT and PE. It is the third most common cardiovascular disorder and affects up to 5% of the population during their lifetime [1], with an annual global incidence rate estimated to be around 115-269 per 100,000 people, and it occurs predominantly in the male elderly population [2]. VTE is triggered by three main factors as described by Virchow in 1856: vascular damage, venous stasis, and hypercoagulability [4]. Different conditions can predispose it, and it has been categorized to have weak (odds ratio (OR) <2), moderate (OR 2-9), or strong risk factors (OR >10) according to the 2019 European Society of Cardiology (ESC) guidelines [5] to guide the patient with decision making as regards thromboprophylaxis according to the frequency of VTE recurrence. In the context of our patient, it has been observed in different studies, such as in the PROLONG study, that VTE occurs more frequently in men than in women (7.4% vs 4.3%) [6]. Other known cardiovascular risk factors present in the patient were obesity (BMI >30), with a hazard ratio of 2.74 and an OR of <2.0 [4, 5], and age ≥ 65 years (OR <2) [5]. Thrombophilia is a moderate risk factor [5], and a family history of VTE conveys an increased risk of VTE. However, only 30% of patients with a family history of VTE will present a positive screening for thrombophilia [7]. Among the conditions that have been associated with an increase in the hyper-coagulant state, the following are listed: (1) clotting factor concentrates (prothrombin complex concentrate, aFVII concentrate, fibrinogen, FVIII, FIX, FXI, etc.); (2) high clotting factor levels (congenital or acquired); (3) administration of anti-fibrinolytics (anti-activators: tranexamic acid, etc.; anti-plasminogen Trasylol, etc.); (4) deficiency of antithrombin, Proteins C and S, FV or FII polymorphisms, hypofibrinolysis, etc.; (5) high-lipid diet (FVII activation); (6) heparin-induced thrombocytopenia, antiphospholipid antibody syndromes; (7) increased platelet number and activation; and (8) drugs. In the same way, different pathologies have been associated with the prothrombotic state, such as (1) a family history positive for thrombosis; (2) hypercoagulable state; (3) neoplastic diseases; (4) cardiac failure; (5) myeloproliferative disease; (6) old age, pregnancy; (7) obesity; (8) surgery; (9) immobilization, trauma; and (10) sepsis [8]. All these risk factors were evaluated and ruled out during the follow-up of the patient, as additional pathologies that triggered the thromboembolic event.

SC is a transitorily cardiovascular condition, described since 1990, found predominantly in postmenopausal women produced after emotional or physical stress, which features signs and symptoms of acute coronary syndrome [3,9]. The pathophysiological mechanisms related to SC remain unclear [9]. Different possible causes have been proposed including sympathetic hyperexcitation, coronary vasospasm, and microvascular disorders [10]. SC has also been related to some critical complications such as heart failure, arrhythmias, left ventricular outflow obstruction, and thromboembolic events [11]. Regarding thromboembolic events, their incidence and clinical significance have not yet been established, and only a few isolated case reports have been reported, which have been associated to long-distance travel [12] and acute pyelonephritis [13]. It has been related to the improvement of left ventricular wall motion abnormalities in SC [14], which promotes the release of intraventricular thrombus into the peripheral bloodstream initiating an embolic event. This has an incidence in at least 5% of the patients [11]. In our patient’s case, this event could have been the trigger of the thromboembolic event, given that complete left ventricular recovery can occur within a few days or can take several weeks [15].

Symptoms and clinical signs are nonspecific. In most cases (up to 25%), PE is suspected in a patient with dyspnea, chest pain, pre-syncope, syncope, or hemoptysis. In some cases, PE may be asymptomatic or discovered incidentally during diagnostic workup for another disease [2,5]. Faced with these situations, the first step in the diagnosis of suspected VTE are pre-test probability assessment for PE, which have been created to avoid unnecessary tests in patients. The Wells and Geneva scores stand out here, as the most used scores by the medical community [7]. However, the proportion of patients with confirmed PE can be expected to be 10%, 30%, and 65% in the low-probability, moderate-probability, and the high-probability categories, respectively [5]. Once the pre-test tests have been carried out, the therapeutic decision can be complemented with other paraclinical tests such as D-dimer (DD) testing and CTPA. DD is a helpful diagnostic tool and has a negative predictive value if the DD test is high, and a normal DD level makes acute PE or DVT unlikely [5,7]. DD concentrations increase with age, and specificity can be improved with an age-adjusted cut-off (age x 10 ng/ml, for patients aged >50 years) [5]). This approach increased the number of patients in whom PE could be excluded without additional diagnostic imaging [7]. CTPA is the gold standard choice for PE imaging; it has high sensitivity and specificity (83% and 96%, respectively) and allows us to visualize pulmonary vasculature adequately and directly [5].

The treatment of PE aims to prevent thrombus extension and embolization, so risk stratification has been used to identify patients with a low short-term mortality risk to select for outpatient management (Pulmonary Embolism Severity Index (PESI)) [7]. The mainstay of treatment is anticoagulation therapy, and the availability of direct oral anticoagulants (DOACs) has simplified outpatient treatment [16]. DOACs has been developed with specific mechanisms, allowing a wider therapeutic window than warfarin, taking a fixed dose without monitoring; three drugs belong to this family (dabigatran, rivaroxaban, and apixaban) [17]. DOACs has shown non-inferiority compared to traditional therapeutic anticoagulation, even showing a significant decline of bleeding risk [16]. The current guidelines [5] recommend that the specific durations of anticoagulation therapy depend on the risk of recurrence in individual patients; several risk-scoring algorithms have been designed to evaluate it, such as the Vienna prediction model, HERDOO2 (hyperpigmentation, edema, or redness in either leg; D-dimer level ≥250 μg/L; obesity with body mass index ≥30; or older age, ≥65 years) score, and DASH tool. In the same way, the risk of bleeding must be considered to optimize the duration of treatment in patients receiving oral anticoagulation treatment, which we find through the VTE-bleed model, HAS-BLED, OBRI, and RIETE scores [5,16]. In patients who are candidates for an extended anticoagulant treatment, as was the case with our patient, studies (EINSTEIN CHOICE [18] and AMPLIFY EXT trial [19]) that validate the use of rivaroxaban and apixaban in reduced doses have been carried out, showing adequate results and low risk of bleeding [5].

The recurrence of PE is influenced by different factors like malignancy [20], male gender (2.2-fold higher risk) [21], and persistently high levels of DD (8.9% annual risk of recurrence) [22]. The rate of recurrence in patients with a history of a previous DVT or PE is at a lifetime increase risk; however, the anticoagulation therapy reduced it by about 80-85% [6]. Rodger et al. [23] describe that in the first year after discontinuing anticoagulation, the rate of recurrent VTE was 10.3 and 3.3 events per 100 patient-years for DVT and PE, respectively. Baglin et al. [24] show that after anticoagulation withdrawal, the five-year cumulative rate of recurrence was 10.6% and 3.1-fold higher in patients with PE; and Pengo et al. [25] showed a rate of recurrence of 11.3 per 100 patient-years in patients with elevated DD levels after a month of anticoagulation treatment discontinuation.

## Conclusions

In conclusion, we described an interesting case of massive PE related to heart failure secondary to SC, which might play a pathophysiological role. Currently, there have been reports of cases in which PE triggered SC, from which it can be inferred that in our case, the pathophysiology of SC might have caused a heart failure with preserved EF in our patient, which caused a decrease in the functional class, indirectly causing a favorable state for VTE.

Also, the optimal duration of anticoagulation beyond the initial three months after the first episode of PE remains uncertain, and the decision after this period should be individually tailored according to the risk of recurrent VTE and balanced against the risk of bleeding.
